# Ferroptosis and mitochondrial dysfunction in acute central nervous system injury

**DOI:** 10.3389/fncel.2023.1228968

**Published:** 2023-08-09

**Authors:** Wenxue Dong, Fanghe Gong, Yu Zhao, Hongmin Bai, Ruixin Yang

**Affiliations:** ^1^Department of Neurosurgery, General Hospital of Southern Theatre Command of PLA, Guangzhou, China; ^2^School of Medicine, Xizang Minzu University, Xianyang, China

**Keywords:** ferroptosis, acute central nervous system injury, traumatic brain injury, spinal cord injury, mitochondrial homeostasis

## Abstract

Acute central nervous system injuries (ACNSI), encompassing traumatic brain injury (TBI), non-traumatic brain injury like stroke and encephalomeningitis, as well as spinal cord injuries, are linked to significant rates of disability and mortality globally. Nevertheless, effective and feasible treatment plans are still to be formulated. There are primary and secondary injuries occurred after ACNSI. Most ACNSIs exhibit comparable secondary injuries, which offer numerous potential therapeutic targets for enhancing clinical outcomes. Ferroptosis, a newly discovered form of cell death, is characterized as a lipid peroxidation process that is dependent on iron and oxidative conditions, which is also indispensable to mitochondria. Ferroptosis play a vital role in many neuropathological pathways, and ACNSIs may induce mitochondrial dysfunction, thereby indicating the essentiality of the mitochondrial connection to ferroptosis in ACNSIs. Nevertheless, there remains a lack of clarity regarding the involvement of mitochondria in the occurrence of ferroptosis as a secondary injuries of ACNSIs. In recent studies, anti-ferroptosis agents such as the ferroptosis inhibitor Ferrostain-1 and iron chelation therapy have shown potential in ameliorating the deleterious effects of ferroptosis in cases of traumatic ACNSI. The importance of this evidence is extremely significant in relation to the research and control of ACNSIs. Therefore, our review aims to provide researchers focusing on enhancing the therapeutic outcomes of ACNSIs with valuable insights by summarizing the physiopathological mechanisms of ACNSIs and exploring the correlation between ferroptosis, mitochondrial dysfunction, and ACNSIs.

## Introduction

Acute central nervous system injury (ACNSI) is the result of sudden damage to the brain or spinal cord due to different factors, including stroke, traumatic brain injury (TBI), and spinal cord injury (SCI) (Shein and Shohami, 2011). ACNSI is a prevalent condition that poses significant risks to human health and life (Shein and Shohami, 2011). This condition is marked by elevated occurrence and death rates, unfavorable treatment results, and may lead to significant impairments and societal impact (Shein and Shohami, 2011). ACNSI involves different types of cell death, such as apoptosis, necrosis, autophagy, and ferroptosis, which contribute to its onset and progression (Walker et al., 2009; El Sayed and Ghoneum, 2020; Bai and Bian, 2022; Sun et al., 2022). Ferroptosis, a form of programmed cell death, differs from other apoptosis as it relies on iron and the buildup of lipid peroxides and their byproducts (Zhang C. et al., 2022). Ferroptosis is intricately linked to various biological processes, encompassing cellular iron levels, metabolism of amino acids and polyunsaturated fatty acids, glutathione (GSH), and biosynthesis of phospholipids (Balihodzic et al., 2022; Zhang C. et al., 2022). The role of this procedure is vital in various illnesses, such as neurologic conditions, malignancies, and ischemia-reperfusion damage (Wang Y. et al., 2020; Jiang et al., 2021). Recent evidence suggests that ferroptosis also plays a significant role in ACNSI (Lei et al., 2020). Following an ACNSI occurrence, there is a rise in intracellular iron levels, which results in heightened lipid peroxidation and subsequent initiation of ferroptosis (Xie et al., 2016). In addition to impacting the viability of neurons, ferroptosis also has an impact on the functionality of glial cells, vascular endothelial cells, and immune cells (Wang W. et al., 2019; Cui et al., 2021; Gao et al., 2022). Moreover, ferroptosis is closely associated with neurological functional deficits, neurodegenerative changes, neuroinflammatory responses, and blood-brain barrier (BBB) damage following ACNSI (Yu J. et al., 2020; Dong et al., 2022; Wang X. et al., 2022). Hence, it is crucial to investigate the mechanisms and functions of ferroptosis in ACNSI and create potent inhibitors or regulators of ferroptosis to prevent and treat ACNSI and its associated complications. The main objective of this article is to thoroughly examine the mechanisms and approaches for treating ferroptosis in ACNSI, specifically emphasizing the advancements in targeted mitochondrial therapy. The results of this investigation will offer valuable perspectives for subsequent studies and aid in the formulation of efficient treatment strategies for ACNSI.

## ACNSI classification and their pathological physiology

ACNSI refers to sudden and unexpected damage to the brain or spinal cord, often caused by trauma, infection, disease, or the administration of certain drugs or chemicals (Slovinska et al., 2022). ACNSI is a frequent reason for mortality or enduring impairment (Shein and Shohami, 2011). ACNSI can be classified into primary and secondary injuries, depending on the mechanical stress situation at the site of injury (Jarrahi et al., 2020). The primary injuries occurs when tissues and blood vessels are directly injured, leading to the death of neurons and glial cells, damage to axons, and hemorrhagic or ischemic injuries due to problems with blood vessels (Ruhatiya et al., 2020). This includes brain contusion, intracranial hemorrhage, brain infarction, axon dysfunction, and other direct injuries caused by pathogens (Lutton et al., 2017; Li Z. et al., 2023). The secondary damage encompasses a range of intricate pathophysiological processes initiated by the initial injury, encompassing excitotoxicity in neurons, activation of neuroglial cells and neuroinflammation, disruption of the BBB, brain swelling, injury caused by ischemia-reperfusion, oxidative stress, and apoptosis (Wang D. et al., 2020; Zhang et al., 2023). Primary and secondary injury interact with each other and jointly influence the clinical outcome of ACNSI.

TBI is an intricate neurological condition caused by physical impact that results in harm to brain function or tissue (Lutton et al., 2017). In addition to 60 million people suffering TBI every year, about half of the world population has suffered one or more TBI over the course of their lifetime (Tanaka and Zhang, 2022). The intricacy of TBI becomes apparent through its various injury mechanisms, which include rapid acceleration/deceleration, direct impact, penetrating injury, and blast waves (Lutton et al., 2017). The outcome is the occurrence of physical harm to structures within the skull and direct harm to the brain tissue, which encompasses hematomas, contusions, diffused axon injury (DAI), and lacerations (Ellis et al., 2016). The primary cause of secondary brain injury is primarily due to heightened levels of excitatory neurotransmitters, generation of reactive oxygen species (ROS), malfunctioning mitochondria, and the release of proinflammatory cytokines (Glotfelty et al., 2019; Lorente et al., 2019; Stelmashook et al., 2019). These factors collectively contribute to the harm and potential demise of neuronal cells. After experiencing additional harm to the nerve tissue, there is a possibility of production of cerebral edema, increased intracranial pressure (ICP), impairment of BBB, and alterations in cerebrovascular reactivity (Lutton et al., 2017). Although there is a clear necessity for efficient and all-encompassing therapies to enhance results and minimize long-lasting impacts, there is currently no specialized and effective treatment for brains that have suffered traumatic injuries. Hence, it is imperative to prioritize the advancement of novel therapies for TBI.

Stroke is a cerebrovascular disease encompassing both hemorrhagic and ischemic strokes. This happens when the blood vessels in the brain are blocked or ruptured, resulting in a sudden decline in brain function (O’Reilly et al., 2014). Intracerebral hemorrhage (ICH) occurs in approximately 10–20% of cases, causing severe disability and, in some instances, death (Hanley et al., 2013). Brain injury following ICH involves primary injury, caused by direct compression and stimulation of the hematoma, and secondary injury, caused by ischemia and hypoxia in the penumbra surrounding the hematoma (Peng et al., 2017). On the contrary, cerebral infarction is a type of brain injury caused by the blockage of blood vessels in the brain. Hypoxia leads to irreversible necrotic death of neurons, glial cells, and vascular endothelial cells in the ischemic region, which is known as the primary injury (Zhou and Huang, 2019). The primary injury can also cause additional harm, such as excitatory neurotoxicity, oxidative stress, inflammation, and cell apoptosis, resulting in expansion of ischemic regions and neurological dysfunction (Dhungana et al., 2017). In the past, stroke treatment has typically included the utilization of oral interventions like tranexamic acid (TXA) and intravenous administrations of edaravone, which have produced disappointing outcomes (Sprigg et al., 2018; Uchida et al., 2022). To effectively treat stroke, it is necessary to protect the well-being of brain tissues and facilitate the healing process in the surrounding and infarcted areas.

In addition, the ACNSI encompasses infective meningitis where microbial pathogens directly invade the central nervous system (CNS) tissues, leading to inflammation and harm (Koelman et al., 2019). The CNS is at significant risk from bacterial meningitis, which could result in fatal outcomes. Annually, the three predominant types of bacteria, namely *Meningococci*, *Haemophilus influenzae*, and *Streptococcus pneumoniae*, contribute to an estimated 1,000,000 cases of meningitis (Chanteau et al., 2006). These bacteria are responsible for causing meningococcal meningitis. Furthermore, meningitis can also be caused by mold, yeast, and fungi that have the ability to change their shape (Davis et al., 2020). The opportunistic infection caused by fungal pathogens, particularly *Cryptococcus neoformans*, known as cryptococcal meningitis (CM), can result in fatality (Sridhar et al., 2021). Despite receiving appropriate antibiotic treatment, mortality remains a significant concern in cases of meningitis.

Different levels and degrees of neurological dysfunction that result from external or internal factors affecting the spinal cord are included in SCI. Direct force applied to the spinal cord causes the primary injury, resulting in the rupture of cell membranes, vascular harm, bleeding, localized lack of blood supply, and embolism (Zavodska et al., 2018). After the primary injury, a secondary injury takes place. The local area experiences the liberation and buildup of a substantial amount of catecholamine neurotransmitters, resulting in microvascular spasm, reduced blood flow, heightened permeability of blood vessels, rupture of small veins, and secondary hemorrhagic necrosis (Liu et al., 2021). SCI causes extensive neuronal demise and axonal disturbance, leading to a state where the functionality of the spinal cord is modified either temporarily or permanently (Chen et al., 2020). Due to the limited regenerative capabilities of the spinal cord, SCI frequently results in enduring impairment to the patient sensory and motor functions (Xi et al., 2020). As a result, SCI continues to be one of the foremost unresolved obstacles in the field of medicine. People who have SCI go through both psychological and physical distress, while medical professionals and patients face significant financial hardships.

## Ferroptosis and mitochondrial dysfunction

Cell death via ferroptosis is distinct from other cell death types. The origin of its name is derived from the iron ions and the lipid peroxides that are linked to it (Ye et al., 2021). In ferroptosis, the cell lipid bilayers undergo oxidation and degradation, leading to the build-up of peroxides and oxidative stress within the cellular environment (Fujiki et al., 2019; Wang T.-X. et al., 2019; Wu Y. et al., 2020). This eventually leads to cell death. The presence of ferroptosis is closely connected to cellular elements like GSH metabolism, lipid metabolism, and iron metabolism. In particular, the build-up of lipid peroxides and the depletion of GPX4 play a crucial role in ferroptosis (Fujiki et al., 2019; Miao et al., 2022; Zhong et al., 2022). Research on ferroptosis offers fresh insights into the mechanisms of cellular demise and unveils innovative therapeutic pathways for delaying or thwarting the progression of diseases (Chen et al., 2022).

There are several mechanisms by which excess iron results to cellular damage. The Haber Weiss reaction can be catalyzed by iron, meaning it has the ability to produce hydroxyl radical (OH) by combining the superoxide anion and hydrogen peroxide, which can cause harm to lipids, nucleic acids, and proteins (Azad et al., 2008; Jabłońska-Trypuć et al., 2017; Lewandowska et al., 2019; Scott et al., 2021). Iron can also bind to transition metals, forming non-biological iron-sulfur clusters. These clusters can transfer electrons and increase ROS in the mitochondria (Chen et al., 2018; Nakamura et al., 2021). Iron can affect neurotransmitter metabolism and release, leading to excessive accumulation or depletion of neurotransmitters such as dopamine, norepinephrine, and serotonin that can interfere with neural signal transmission (Zhou and Tan, 2017; Kletetschka et al., 2021).

Ferroptosis and mitochondria are closely related. In the cell, mitochondria are the primary energy producers and are crucial for controlling cell death (Perez-Pinzon et al., 2012; Law et al., 2018). Mitochondrial dysfunction and damage resulting in ferroptosis, can be caused by the abnormal buildup of iron ions as observed in several studies (Dong et al., 2022). Ferroptosis displays unique morphological features, such as reduced mitochondria and increased density of mitochondrial membrane, leading to impaired mitochondrial function (Wang T.-X. et al., 2019). Iron ions can affect mitochondrial function in several ways, including inhibition of electron transport chain enzyme activity, increased ROS production, mitochondrial membrane peroxidation, and loss of inner membrane potential (Hu et al., 2016; Jiang et al., 2020; Jing et al., 2021). The inhibition of GPX4 can occur as a consequence of ferroptosis, either through direct or indirect means (Cao and Dixon, 2016). Consequently, this can result in harm to the intracellular antioxidant mechanism and lead to the buildup of ROS in mitochondria, ultimately causing cellular impairment (Dong et al., 2020). Due to malfunctioning mitochondria, the impairment of GSH, a GPX4 substrate is exacerbated, resulting in the accumulation of iron overload and ROS in membrane lipids (Wang et al., 2022b). Moreover, ferri-liposomes possess the capability to trigger apoptosis and ferroptosis through the liberation of citric acid iron oxide nanoparticles (IONPs-Ac) within the cellular cytosol. This action enhances mitochondrial dysfunction, ultimately resulting in the onset of apoptosis/ferroptosis (de Souza et al., 2021). The formation of mitochondrial dysfunction in CNS disease has been identified as triggered by ferroptosis, according to recent discoveries on cell death mechanisms (Krabbendam et al., 2020).

## Ferroptosis and mitochondrial dysfunction in TBI

TBI has the ability to trigger a recently discovered type of cellular demise called ferroptosis, which is distinguished by significant buildup of iron and heightened oxidative stress in the impacted area (Cheng et al., 2023). Mitochondria, which are essential controllers of cellular destiny, have a vital function in the progression of both TBI and ferroptosis (Shen et al., 2019). Nonetheless, the exact function of mitochondria in ferroptosis is still a subject of debate. Certain academics have put forth proof indicating that malfunction in mitochondria may amplify the generation of ROS, consequently intensifying the incidence of ferroptosis (Shojaie et al., 2020). Furthermore, TBI is recognized to cause long-lasting cognitive and motor disabilities, and there is a lack of effective therapies for these individuals. Nevertheless, with the application of our existing knowledge about ferroptosis, we can develop innovative treatment approaches focused on addressing the crucial molecules and pathways involved in this mechanism. These strategies promise in reducing neuronal damage and enhancing functional recovery following TBI.

Neuronal membrane lipids are harmed by ferroptosis, which is a form of cellular demise caused by oxidative stress and lipid peroxidation. ROS initiates ferroptosis-mediated oxidative stress, which is the primary mechanism of ferroptosis and results in lipid peroxidation (Yang et al., 2022). Mitochondria are both major sources and targets of ROS, which play a vital part in starting and carrying out ferroptosis (Shojaie et al., 2020). Multiple pieces of evidence substantiate the role of mitochondria in ferroptosis. In mouse embryonic fibroblasts (MEF) and HT-22 hippocampal cells (HT-22) cells, the production of mitochondrial ROS is significantly enhanced by erastin and Ras selective lethal 3 (RSL3) (Xia et al., 2016; Fang et al., 2017; Rodríguez-Graciani et al., 2022). In addition, the reduction of ferroptosis can be achieved by using mitochondria-specific ROS scavengers like mitoquinone (MitoQ), XJB-5-131, and triphenylphosphonium chloride (Mito-TEMPO) (Park et al., 2017; Broome et al., 2018; Guigni et al., 2018). Following TBI, excessive glutamate release and impaired uptake result in excitotoxicity, leading to calcium overload and ROS generation in neurons (Shen et al., 2019). These factors trigger mitochondrial dysfunction, including the decline in membrane potential, the opening of the mitochondrial permeability transition pore, the liberation of cytochrome c (Cyt c), and the initiation of caspases (Wang P. et al., 2022). The after-effects of trauma lead to situations where the reduced usual number of mitochondria face higher metabolic requirements, leading to a significant increase in the production of ROS (Wang P. et al., 2022). Excitotoxicity, free iron, and interactions among ROS contribute to the excessive production of ROS (Lee et al., 2017; Liu et al., 2023). Mitochondrial damage exacerbates the ferroptosis process by inducing additional production of ROS and abnormal release of ROS (Taeubert et al., 2021). To elaborate, CNS injury causes mitochondrial damage, affecting the balance of mitochondrial dynamics (Chang et al., 2019). The disruption in mitochondrial dynamics results in decreased quantity, integrity, and performance of mitochondria, consequently hindering their capacity to produce energy and withstand oxidative stress (Di Pietro et al., 2017). Consequently, neurons become more susceptible to ferroptosis. Hence, focusing on the impairment of mitochondria presents a hopeful approach to hinder or counteract the consequences of TBI and ferroptosis.

The research carried out by Gao et al. (2019) demonstrated that the suppression of mitochondrial function effectively inhibited ferroptosis induced by cysteine deprivation. A substantial rise in mitochondrial metabolism occurred when cysteine levels were depleted, leading to an increased utilization of GSH. As a result, there was an ensuing increase in the generation of lipid ROS, ultimately resulting in ferroptosis (Gao et al., 2019). By increasing GSH levels, reducing oxidative stress, and regulating neurotransmitter systems, among other mechanisms, the inclusion of N-acetyl cysteine (NAC), a cysteine supplement, has the capacity to improve cognitive and behavioral functions after TBI (Huang et al., 2018). The decrease in cysteine concentrations after TBI may be due to its use in fighting against oxidative stress, inflammatory reaction, neuronal demise, and imbalances in neurotransmitters during pathological circumstances (Chen et al., 2018). Although active mitochondria may enhance ferroptosis, it has been observed that impaired mitochondrial function alone is not enough to effectively alleviate this process (Gao et al., 2019; Pandur et al., 2019). This may be caused by other processes that produce lipid ROS, which are quickly intensified by the Fenton reaction, ultimately leading to complete ferroptosis cell demise (Gao et al., 2019; Zhang et al., 2021). Nonetheless, the precise role of mitochondrial function in ACNSI necessitates further substantial evidence to substantiate its mechanisms.

The disparity between an overabundance of iron ions being consumed and released leads to an accumulation of iron within cells, ultimately leading to the production of extremely reactive free radicals OH via the Fenton reaction, thereby resulting in heightened oxidative harm. The activation of the Fenton reaction occurs when cells take in an excess of ferrous ions, resulting in lipid peroxidation (Jing et al., 2021). The presence of this mechanism plays a crucial role in triggering ferroptosis, thus emphasizing the important outcomes resulting from the excessive generation of lipid peroxides and the initiation of free radicals (Jing et al., 2021). Following TBI, axons in the brain white matter become more vulnerable to damage (Han et al., 2015). The main component of DAI is the physical breakage of the axonal cytoskeleton, which disrupts the transportation of axons and leads to swelling and breakdown of proteins (Lazarus et al., 2015). Secondary trauma pertains to subsequent molecular and chemical inflammatory responses, like the liberation of hemoglobin and iron, impaired brain metabolism, and cerebral blood flow (Wei et al., 2012). These reactions further trigger neuroinflammatory processes, oxidative stress, glutamatergic excitotoxicity, and mitochondrial malfunction, ultimately resulting in further brain damage (Lipponen et al., 2019; Liao et al., 2020). The BBB is compromised by primary and secondary injuries caused by TBI, enabling iron to enter brain tissue and create deposits of iron (Yauger et al., 2019). The presence of iron initiates the Fenton reaction, resulting in the production of extremely reactive OH free radicals, which in turn leads to the occurrence of oxidative stress and lipid peroxidation (Zhu et al., 2021). These processes represent key features of ferroptosis. Moreover, TBI also disrupts the balance of iron uptake, storage, and efflux, resulting in intracellular iron overload and dysfunction (Cheng et al., 2023). It is well-established that ROS, including superoxide anion free radicals and OH, can contribute to brain injury in TBI patients (Shi et al., 2018). The overabundance of iron and malfunction increase the activation of heme oxygenase-1 (HO-1), subsequently enhancing the onset of ferroptosis (Chen et al., 2023). Furthermore, potential elements that may contribute to brain iron metabolism disorder could include dysfunction of mitochondria and lysosomes. Under pathological conditions of TBI, the MCU protein situated on the inner membrane of mitochondria has the ability to convey significant quantities of calcium ions into the mitochondria, causing disruption to their regular operation (Zhang L. et al., 2019). The rapid transportation of calcium ions into mitochondria by MCU is accompanied by the simultaneous transportation of significant amounts of iron into the mitochondrial matrix, which could potentially result in a disorder of iron homeostasis (Zhang L. et al., 2019). The overabundance of iron triggers the generation of ROS through the Fenton reaction, worsening the impairment of mitochondria and creating an ongoing destructive cycle that intensifies iron-triggered cellular demise (Jing et al., 2021). Regulating iron metabolism, scavenging free radicals, and safeguarding the normal functioning of mitochondria are potential strategies to prevent iron-induced cell death following TBI.

By altering the ferroptosis pathway, TBI could be reduced in the following ways (Table 1).

**TABLE 1:** Therapeutic strategies targeting ferroptosis in TBI.

Treatment	Mechanism	Effects/Outcomes
Epifriedelinol (EFD)	Enhances serum cytokine levels and diminishes oxidative stress	Mitigates secondary damage after TBI
Folic acid (FA)	Regulates neuroinflammation caused by iron ptosis through inhibition of Nrf2/GPX4 axis activation	Prevents ferroptosis; reduces brain edema and neuroinflammation
Ferrostain-1 (Fer-1)	Inhibits ferroptosis	Enhances potential of mitochondrial membrane, ATP synthesis, and decreases ROS levels
Liproxstatin-1 (Lip-1)	Decreases brain swelling and neuroinflammation by inhibiting internal lipid peroxidation	Protects cells against harm caused by oxidative stress
Cerium oxide nanoparticles (CeONPs)	Reducing oxidative stress and mitochondrial impairment	Potential in treating disorders associated with mitochondrial dysfunction and reduces harmful impacts of TBI
Iron chelators (deferoxamine, deferasirox, deferiprone)	Reduce accessibility of intracellular free iron, prevent iron-triggered oxidative reactions	Improve TBI outcomes, inhibit ferroptosis
Superoxide dismutase (SOD)	Prevents production of OH	Enhances outcome of TBI, reduces brain damage
N-acetylcysteine (NAC)	Remove excess oxidative free radicals, restore GSH levels, decrease mitochondrial dysfunction	Restore cellular function, alleviate inflammation and cognitive dysfunction
Antioxidant peptide SS-31	Reverses mitochondrial dysfunction, decreases ROS levels, protects against decline in SOD activity, hinders ferroptosis	Relieves neurological impairments, brain water accumulation, DNA harm, and neuronal cell death

Reducing oxidative stress is regarded as a crucial goal in the management of TBI. Epifriedelinol (EFD) has demonstrated its ability to mitigate secondary damage in rats with TBI by enhancing serum cytokine levels and diminishing oxidative stress (Li et al., 2018). The *Nrf2* gene expression, accountable for the response to oxidative stress, has been discovered as a potential mechanism for averting ferroptosis. Research has demonstrated that the use of folic acid (FA) therapy can regulate neuroinflammation caused by iron ptosis through the inhibition of *Nrf2/GPX4* axis activation (Wang C. et al., 2022). Noteworthy, Wu et al. (2018) Discovered that Ferrostain-1 (Fer-1), a substance that inhibits ferroptosis, has the ability to successfully restore mitochondrial malfunction. Evidence suggests that Fer-1 enhances the potential of the mitochondrial membrane and the synthesis of Adenosine Triphosphate (ATP), while also decreasing ROS levels (Wu et al., 2018). This has also been actively used in subarachnoid hemorrhage (SAH) to protect against brain edema and neuroinflammation (Gao et al., 2022). Liproxstatin-1 (lip-1), an additional iron inhibitor, is employed to decrease brain swelling and neuroinflammation. It hinders internal lipid peroxidation and safeguards cells against harm caused by oxidative stress (Gao et al., 2022). Furthermore, considering the link between increased oxidative stress and mitochondrial impairment as underlying causes for the neurological outcomes of TBI, the utilization of cerium oxide nanoparticles (CeONPs) could potentially be effective in reducing the harmful impacts of TBI (Bailey et al., 2020). These discoveries have important implications in the treatment of disorders associated with mitochondrial dysfunction.

Inhibiting the presence of unbound iron ions and free radicals is a feasible strategy to impede ferroptosis in TBI. The use of iron chelation treatment has shown promise in improving the negative consequences of TBI. Iron chelators, such as deferoxamine (DFO), deferasirox (DFX) and deferiprone (DFP), effectively limit the accessibility of intracellular free iron, thereby reducing the likelihood of iron-triggered oxidative reactions (Votavova et al., 2021; Zhou et al., 2022). Recent investigations indicate that DFO treatment also inhibits the emergence of free radicals and prevents ferroptosis (Zhou et al., 2022). Besides the use of iron chelation, superoxide dismutase (SOD) has been recognized as a hopeful strategy to enhance the outcome of TBI (Kobeissy et al., 2016). The function of SOD is to prevent the production of OH, thereby reducing brain damage (Lv et al., 2016). To decrease the occurrence of ferroptosis, cells make use of antioxidants like GSH and NAC to remove an excess of oxidative free radicals. Studies have shown that the sole administration of NAC systemically can restore GSH levels and decrease mitochondrial dysfunction in rats with TBI (Gomez et al., 2013; Du et al., 2016). On the other hand, when NAC is combined with minocycline, it can alleviate inflammation and cognitive dysfunction in TBI rats (Margulies et al., 2016). Several research studies have indicated a favorable pattern in the utilization of antioxidant treatment, and traditional antioxidants can function as supplementary therapy for TBI. Nevertheless, these traditional antioxidants lack specificity in their actions, frequently requiring high levels to attain desired therapeutic outcomes. Effective mitochondrial targeting requires the crucial delivery of antioxidants specifically to the mitochondria. Administration of the antioxidant peptide SS-31, 30 min after mild TBI, significantly reverses the mitochondrial dysfunction caused by TBI and improves secondary brain injury (Shen et al., 2019). SS-31 directly decreases the level of ROS in brain tissue, protects against the decline in SOD activity, and hinders ferroptosis (Righi et al., 2013). As a result, it relieves neurological impairments, brain water accumulation, DNA harm, and neuronal cell death (Shen et al., 2019). Furthermore, the peroxisome proliferator-activated receptor γ (PPARγ) signaling pathway is crucial for cellular function. Enhancing the cell antioxidant defense mechanism is one of the ways it helps to facilitate the expression and activity of GPX4 in cells (Wang M.-J. et al., 2023). Through ongoing research and innovative treatment techniques like these, healthcare professionals can aspire to mitigate the ferroptosis caused by TBI and improve patient outcomes.

## Ferroptosis and mitochondrial dysfunction in non-traumatic brain injury

### Stroke

Stroke is a neurological disorder that mainly happens when brain cells are harmed and die because of a lack of oxygen and inadequate blood flow in the affected region. This occurs due to the blockage of arteries in the brain. There are two types of stroke: ischemic and hemorrhagic. Globally, it ranks second in terms of mortality and is the main reason for fatalities in China, which carries the highest burden of stroke cases worldwide (Mei et al., 2020).

In instances of hemorrhagic stroke and advanced cerebral ischemia, Ferroptosis has been identified. The presence of iron-rich blood and ferritin-rich brain tissue is a result of the compromised BBB and the primary brain injury caused by direct compression and stimulation of the hematoma (Liu et al., 2019). Following ICH, it takes a few weeks for brain heme iron levels to significantly increase, while levels of transferrin (TF), transferrin receptor (TFR), and ferritin (FT) in the brain take a few days to rise (Wu et al., 2003). The disproportionate rise in proteins responsible for iron handling and excessive accumulation of iron can result in harmful reactions that cause damage to the brain (Park et al., 2015; Gill et al., 2018). The release of iron from the hematoma following ICH could potentially lead to oxidative stress, perihematomal edema, and an elevation in ROS levels, ultimately leading to ferroptosis (Zhang H.-Y. et al., 2022).

Following the onset of cerebral hemorrhage, the surrounding region of the hematoma experiences secondary brain damage due to ischemia and lack of oxygen. Likewise, cerebral infarction leads to primary damage and triggers additional mechanisms of injury, including excitotoxicity, oxidative stress, inflammation, and apoptosis (Dhungana et al., 2017). These mechanisms play a role in the enlargement of the ischemic region and subsequent impairment of neurological function. Children with hypoxic-ischemic encephalopathy often exhibit iron deposition in the basal ganglia, thalamus, and periventricular white matter (Dietrich and Bradley, 1988). The presence of iron in the brain tissue could be due to the breakdown of the BBB, which allows iron from the blood to enter the brain. The function of iron transport and storage proteins is impacted by hypoxia, which leads to an increased susceptibility of neurons to iron (Gill et al., 2018). The Fenton reaction is responsible for the aggravation of lipid peroxidation-induced ferroptosis due to an excess of iron, leading to increased infarct sizes (Castellanos et al., 2002; Jing et al., 2021). Furthermore, in a mouse model of ischemic stroke, it was observed that free radicals and an excess of iron resulted in the prolonged upregulation of transferrin receptor 1 (*TFR1*), which subsequently led to an elevation in peripheral iron absorption, oxidative stress, and the demise of neurons (Yu Y. et al., 2020). Moreover, diminished GSH levels, which are evident in both individuals with stroke and animal models of middle cerebral artery occlusion (MCAO), additionally amplify ferroptosis (Liu et al., 2020). Cerebral ischemia-reperfusion injury (CIRI) exhibits an imbalance in lipid and amino acid metabolism. For example, the levels of malondialdehyde (MDA) and nitric oxide (NO) are increased, whereas the levels of SOD and GPX4 are decreased in mouse models of CIRI and oxygen-glucose deprivation/reoxygenation (OGD/R) cell models (Zeng et al., 2019). Furthermore, an imbalance in the metabolism of amino acids contributes to the promotion of ferroptosis. In rats with middle MCAO, the levels of extracellular glutamate significantly increased. This led to the acceleration of neuronal iron absorption and subsequent cell death due to excitatory toxicity (Shu et al., 2021). This could be attributed to the regulation of glutaminase 2 by the *p53* gene, which triggers ferroptosis when glutaminase increased (Jennis et al., 2016). Furthermore, mitochondria have a vital function in upholding cellular balance and operation, and their impairment is linked to the development of heart and brain disorders (Horvath et al., 2008). In individuals suffering from acute ischemic stroke, plasma analysis indicated an increase in the expression of lncRNA *PVT1* and a decrease in the expression of *miR-214* (Lu et al., 2020). In CIRI mice, the infarct area was reduced and ferroptosis was suppressed by either silencing *PVT1* or increasing the expression of *miR-214* (Lu et al., 2020).

The phenomenon of ferroptosis has been observed to intensify the severity of stroke by inducing oxidative stress and lipid peroxidation in the brain (Table 2), similar to the mechanisms underlying TBI. GSH levels and GPX4 levels have been strongly associated with stroke. Following a stroke, the levels of GSH and GPX4 expression decrease, impeding the prompt elimination of the oxidative stress reaction and worsening ferroptosis (Alim et al., 2019). By activating the transcription factors *TFAP2c* and *Sp1*, Selenium has the potential to enhance *GPX4* expression, offering a possible therapeutic strategy (Alim et al., 2019). Studies have demonstrated that drugs like metformin can safeguard the brain by inhibiting ferroptosis, either by decreasing the production of lipid peroxidation or by elevating GPX4 levels, ultimately reducing hemorrhagic or ischemic brain damage in mice (Abd-Elsameea et al., 2014; Ismail Hassan et al., 2020). This process helps prevent ferroptosis and glutamic acid (Glu) excitotoxicity, which in turn reduces hemorrhagic or ischemic brain damage in mice (Alim et al., 2019). The quantity or activity of LOX-5 has been observed to rise in both ischemic and hemorrhagic strokes, potentially initiating lipid peroxidation and ultimately causing neuronal ferroptosis (Yigitkanli et al., 2017; Karuppagounder et al., 2018). A different research study indicated that the antioxidative characteristics of NAC have the ability to neutralize the harmful effects caused by nuclear LOX-5 and hinder neuronal ferroptosis (Karuppagounder et al., 2018). Extracted from safflower, carthamin yellow, a compound of flavonoid nature, has been found to hinder the buildup of iron and ROS in the rat brain suffering from CIRI. Additionally, it can restore the protein expression levels of ACSL4, TFR1, GPX4, and ferritin heavy chain 1 (Guo et al., 2021). Furthermore, inhibition of Tau was found to protect young animals from ferroptosis during ischemia-reperfusion following ischemic stroke, subsequently suppressing neural damage (Tuo et al., 2017). It is crucial to address oxidative stress and lipid peroxides in individuals who have experienced a stroke in order to avoid additional harm to cells.

**TABLE 2:** Therapeutic strategies targeting ferroptosis in stroke.

Treatment	Mechanism	Effects/Outcomes
Selenium	Activates transcription factors TFAP2c and Sp1, enhances GPX4 expression	Offers a possible therapeutic strategy for stroke
Metformin	Decreases production of lipid peroxidation, elevates GPX4 levels	Safeguards the brain, reduces hemorrhagic or ischemic brain damage
LOX-5 inhibitors	Reduces lipid peroxidation, hinders neuronal ferroptosis	Prevents neuronal ferroptosis in stroke
N-acetylcysteine (NAC)	Neutralizes harmful effects of nuclear LOX-5, inhibits neuronal ferroptosis	Protects against neuronal ferroptosis in stroke
Carthamin yellow	Inhibits accumulation of iron and ROS, restores protein expression levels of ACSL4, TFR1, GPX4, and ferritin heavy chain 1	Reduces oxidative stress and lipid peroxides in stroke
Tau inhibition	Protects against ferroptosis during ischemia-reperfusion, suppresses neural damage	Offers neuroprotection in ischemic stroke

### Meningitis

*Neisseria meningitidis* is a prominent etiological agent of bacterial meningitis, as it colonizes the human pharynx and leads to severe diseases such as meningitis (Harrison, 2006). Moreover, in Africa, CM is the primary cause of adult meningitis (Kwizera et al., 2021). CM is a disease caused by the fungus *Cryptococcus*, which is usually more common in people with weakened immune systems (Kwizera et al., 2021). These pathogens can infect the patient body through inhalation, causing manifestations such as pneumonia. In some cases, *Cryptococcus* can circulate through the bloodstream and reach the brain, causing meningitis (Kwizera et al., 2021; Retchless et al., 2022). The disease presents with a range of symptoms, including headache, fever, nausea and vomiting, and stiff neck, among others (Haidrani, 2016; Bårnes et al., 2018; Lin and Su, 2020). If left untreated, the disease can pose a significant risk to the patient life.

Recent studies have increasingly demonstrated a correlation between meningitis and ferroptosis, yet the precise mechanisms that underlie this association remain unclear. It is hypothesized that ferroptosis may impact the progression of meningitis by inducing iron accumulation and alterations in lipid peroxidation. Individuals with CM have elevated ferritin levels in their cerebrospinal fluid (CSF) (Deisenhammer et al., 1997). Furthermore, the increase in ferritin levels can be considered a sign of meningitis (Campbell et al., 1986). Another study has indicated that iron overload exacerbates experimental CM (Barluzzi et al., 2002). The process of ferritinophagy, which involves the release of iron into the labile iron pool (LIP) through ferritin, has been found to increase ferroptosis sensitivity (Xu et al., 2021). As demonstrated in multiple studies, both the vasculature and brain are susceptible to *C. neoformans*-induced lipid peroxidation. Notably, Hall et al. observed a significant increase in cellular lipid peroxidation levels in rabbits inoculated with *C. neoformans* (Hall et al., 2010).

## Ferroptosis and mitochondrial dysfunction in SCI

An extremely devastating traumatic injury is SCI, characterized by massive neuronal death and axonal disruption (Wang R. et al., 2023). After suffering from SCI, the individual might encounter lasting limitations in both their motor and sensory abilities due to the spinal cord restricted ability to regenerate (Xi et al., 2020). Treating SCI efficiently continues to be one of the most significant medical obstacles to this day (Simard et al., 2010).

Iron overload and ROS accumulation result in SCI-induced downward translocation of motor neuron iron and impaired recovery. By quantifying iron deposition, demyelination, and atrophy over 2 years after SCI, researchers found that cord atrophy and cerebellar loss decreased, and while brain white and gray matter atrophy was sustained, the myelin content in the spinal cord and cortex reduced progressively. As the sustained atrophy in the thalamus progressed, iron deposition was significant. Feng et al. showed that motor cortex microglia respond to iron overload by accumulating ROS, resulting in ferroptosis of motor neurons and thereby hindering recovery following SCI (Feng et al., 2021). The augmented accumulation of iron was confirmed in both rats and individuals with SCI. Furthermore, the use of iron chelators, inhibitors of ROS, and inhibitors of ferroptosis was found to decrease the death of motor neurons and promote the restoration of function. Furthermore, activated microglia were discovered to release NO, which regulates the balance of cellular iron and causes an excessive accumulation of iron in motor neurons (Feng et al., 2021). In a separate investigation, scientists employed a modified Allen method to create a rat model for DFO treatment at the thoracic T10 section, and compared it to a mouse model for SCI. Examination with electron microscopy revealed diminished mitochondria in the SCI cohort. Unlike the SCI group, the DFO group showed significantly better Basso, Beattie, and Bresnahan locomotor scores, lower iron levels, and higher levels of GPX4, xCT, and GSH expression. In addition, the DFO group exhibited increased expression of mRNA for genes associated with ferroptosis, specifically Acyl-CoA synthetase family member 2 (ACSF2) and iron-responsive element-binding protein 2 (IREB2). Additionally, DFO was observed to promote neuronal survival and suppress gliosis (Yao et al., 2019). A distinct investigation discovered that there was an increase in the manifestation of the ferroptosis indicator ACSL4 and the oxidative stress indicator MDA in the subset of individuals who had experienced SCI within 24 h of the incident. Moreover, there was an observed decline in GSH levels throughout this timeframe. The results, when combined with bioinformatics analysis, suggested that the initial day after SCI played a vital role in the advancement of ferroptosis. Through bioinformatics analysis, the study additionally discovered 10 crucial hub genes associated with ferroptosis, specifically *STAT3*, *JUN*, *TLR4*, *ATF3*, *HMOX1*, *MAPK1*, *MAPK9*, *PTGS2*, *VEGFA*, and *RELA*. Following injury, the SCI group displayed increased levels of *STAT3*, *JUN*, *TLR4*, *ATF3*, *HMOX1*, *PTGS2*, and *RELA* mRNA, while *VEGFA*, *MAPK1*, and *MAPK9* mRNA levels were found to be decreased, as confirmed by real-time PCR analysis, in comparison to the sham group, 1 day post-injury. This pattern was not observed in the sham group (Li et al., 2023). DHODH expression and enzymatic activity have been implicated in cancer progression in previous studies. Currently known inhibitors such as leflunomide metabolites and teriflunomide are effective therapeutic agents for the treatment of autoimmune diseases such as rheumatoid arthritis (RA) (Li et al., 2023). DHODH is targeted by immunosuppressive agents and anticancer drugs, which have shown potential in protecting the CNS, promoting neuronal survival, and facilitating axon regeneration (Li et al., 2023). DHODH, located on the outer surface of the inner mitochondrial membrane, plays a crucial role in detoxifying lipid peroxyl radicals in mitochondria by catalyzing the oxidation of dihydroorotate and reducing coenzyme Q (CoQ) to CoQH2 (Lyamzaev et al., 2023). Recent studies have also identified a similar antiferroptotic mechanism involving CoQ reduction by mitochondrial glycerol-3-phosphate dehydrogenase 2 (GPD2) (Lyamzaev et al., 2023). In a study conducted by Li et al. (2023) molecular evidence was provided indicating that DHODH acts as an inhibitor, preventing the activation of molecules associated with ferroptosis. This inhibition leads to a decrease in the production of lipid peroxides and mitochondrial damage, which ultimately results in the reduction of neuronal ferroptosis (Li et al., 2023). While the exact role of DHODH in the other CNS remains unclear, exploring its association with neurodegenerative diseases and iron-dependent cell death in ACNSI provides new possibilities for therapeutic interventions. These opens up a new idea for the relationship between DHODH and iron death in ACNSI.

The therapeutic strategies aimed at reversing or delaying neurological damage following SCI by countering ferroptosis have been achieved through the targeting of the ferroptosis pathway (Table 3). One approach includes utilizing DFO, a compound that binds to iron, leading to an upregulation of GPX4, systemic Xc^–^, and GSH levels. Consequently, this leads to a decrease in excessive iron accumulation and neuronal ferroptosis within the motor cortex following SCI, thus aiding in the restoration of motor function (Zhang et al., 2020). Another method is to utilize the ferroptosis inhibitor Fer-1, which can improve SCI by reducing iron and ROS deposition, thereby inhibiting ferroptosis in oligodendrocytes (Wang F. et al., 2022). Furthermore, an innovative compound named SRS 16-86 has shown promising outcomes in SCI models by enhancing GPX4, GSH, and xCT levels, as well as diminishing lipid oxidation, gliosis, and neuronal harm (Zhang Y. et al., 2019). Proanthocyanidins, which are potent free radical scavengers, have also shown promise in treating nerve damage in SCI. They efficiently decrease the amounts of iron and substances that react with thiobarbituric acid, inhibit the expression of *ALOX15* and *ACSL4*, and enhance the levels of HO-1, NRF2, GSH, and GPX4 (Zhou et al., 2020). Moreover, zinc gluconate has shown the capacity to reduce ferroptosis in SCI by enhancing *NRF2/HO-1* signaling. As a consequence, there is a rise in GSH, SOD, and GPX4 levels, accompanied by a decrease in ROS and lipid peroxides. Zinc gluconate also promotes the healing of injured mitochondria and inflammation, making it a potential future option for promoting both behavioral and structural recovery after SCI (Li et al., 2019). Notably, a study investigating the DHODH inhibitor teriflunomide in a SCI model revealed significant neuroprotective effects, including improved motor function recovery, reduced inflammation, increased neuronal survival, and inhibition of apoptosis, autophagy, mitochondrial dysfunction, and oxidative stress (Li et al., 2023). These evidence that terfenamine may protect Nervous tissue after SCI through various ways.

**TABLE 3:** Therapeutic strategies targeting ferroptosis in SCI.

Treatment	Mechanism	Effects/Outcomes
Deferoxamine (DFO)	Binds to iron, upregulates GPX4, systemic Xc-, and GSH levels	Decreases iron accumulation and neuronal ferroptosis, aids in restoration of motor function in SCI
Ferrostain-1 (Fer-1)	Reduces iron and ROS deposition, inhibits ferroptosis in oligodendrocytes	Improves SCI by inhibiting ferroptosis
SRS 16-86	Enhances GPX4, GSH, and xCT levels, decreases lipid oxidation, gliosis, and neuronal harm	Shows promising outcomes in SCI models
Proanthocyanidins	Decrease iron and substances that react with thiobarbituric acid, inhibit ALOX15 and ACSL4, enhance HO-1, NRF2, GSH, and GPX4 levels	Potentially treats nerve damage in SCI
Zinc gluconate	Enhances NRF2/HO-1 signaling, increases GSH, SOD, and GPX4 levels, decreases ROS and lipid peroxides	Reduces ferroptosis, promotes behavioral and structural recovery after SCI
Teriflunomide	Exhibits neuroprotective effects, improves motor function recovery, reduces inflammation, increases neuronal survival, inhibits apoptosis, autophagy, mitochondrial dysfunction, and oxidative stress	Protects nervous tissue after SCI

## Summary and prospect

Numerous studies have shown the significance of ferroptosis in ACNSI, specifically in relation to secondary injury following ACNSI. In this process, the significant role of mitochondria has been discovered and confirmed by researchers, despite the existence of controversial mechanisms (Wang et al., 2022a). Numerous researches have verified that malfunctioning mitochondria can trigger oxidative stress and additional elements that contribute to ferroptosis and neuronal cell death (Bécquer-Viart et al., 2021). Scientific evidence demonstrates that mitigating ferroptosis has the potential to postpone or even reverse neural damage. Different methods can be employed to accomplish this, including the use of iron chelators, lipid antioxidants, boosting GPX4 activity or stability, elevating GSH levels, or suppressing Ferroptosis Suppressor Protein 1 (FSP1) activity (Singh et al., 2014; Hayano et al., 2016; Serra et al., 2020; Wu L. et al., 2020; Wang F. et al., 2022). Conventional antioxidants typically lack selectivity and require high concentrations for therapeutic effects. Therefore, it is essential to direct antioxidants specifically toward mitochondria in order to achieve a targeted effect on mitochondria. Mitochondrial-targeted therapy involves protective strategies for CNS cells against ferroptosis by improving or replacing damaged mitochondria. One example is DHODH inhibitor, called teriflunomide targets mitochondrial ferroptosis (Malla et al., 2020). Research has also been conducted on nanomaterials for precise delivery of drugs to mitochondria, although there is still a need for accurate regulation of drug release amount and location. The regulation of the dynamics of mitochondria, which involves encouraging the splitting and merging of mitochondria, as well as the removal of damaged mitochondria through mitophagy, holds potential in counteracting ferroptosis. Nevertheless, the regulation of mitochondrial dynamics necessitates the consideration of various elements, including but not limited to timing, dosage, and duration. Moreover, there have been reports on the potential use of mitochondrial transplantation as a remedy for different ailments, providing a chance to combat ferroptosis and reverse neural damage. Nevertheless, additional investigation is necessary to explore the molecular signaling pathways, regulatory elements, and factors that exert influence. The potential for treating ACNSI by targeting mitochondria to counteract ferroptosis is significant. However, further fundamental investigations and clinical experiments are required to confirm effectiveness and security, as well as ascertain the most suitable treatment plan and indications.

Undoubtedly, the process of ferroptosis in instances of ACNSI is a highly debated and promisingly investigated subject. Suppressing ferroptosis offers a promising avenue for treating the prognosis of ACNSI in the future. However, the implementation of these ideas is currently limited to experimental settings, although it is expected that they will eventually be expanded to a broader range of individuals.

## Author contributions

RY and HB designed and coordinated the study. WD and YZ collected the references in related fields and wrote the first draft of the manuscript. FG and RY revised the manuscript. All authors reviewed and approved the final manuscript.
